# Changes in health-related quality of life of Chinese populations measured by the EQ-5D-3 L: a comparison of the 2008 and 2013 National Health Services Surveys

**DOI:** 10.1186/s12955-019-1109-x

**Published:** 2019-03-11

**Authors:** Qiang Yao, Chaojie Liu, Yaoguang Zhang, Ling Xu

**Affiliations:** 1 School of Political Science and Public Administration, Institute of Health Research, Wuhan University, Wuhan, Hubei China; 2 School of Psychology and Public Health, La Trobe University, Melbourne, VIC Australia; 3 Center for Health Statistics and Information, National Health Commission, Beijing, China; 4 Health Human Resources Development Center, National Health Commission, Beijing, China

**Keywords:** Health-related quality of life, EQ-5D-3 L, National Health Services Surveys, China

## Abstract

**Backgrounds:**

The EuroQol Group Five-Dimensional (EQ-5D) instruments have been validated in China for measuring health-related quality of life (HRQoL) and are increasingly being used in health economic studies. However, there is paucity in the literature documenting long-term changes in the EQ-5D results in the Chinese populations. This study aims to identify such changes and their determinants using the EQ-5D-3 L instrument.

**Methods:**

Data were obtained from the National Health Services Surveys in China, which included the EQ-5D-3 L since 2008. We compared the differences between the 2008 and 2013 surveys in the percentage of reported problems, visual analogue scale (VAS) scores, and the EQ-5D-3 L utility index derived from the national value sets. Factors associated with population changes in these EQ-5D results were identified using logistic, linear and Tobit regression models, respectively.

**Results:**

Compared with 2008, reported problems in self-care (3.3% vs 3.1%), usual activities (4.8% vs 4.6%) and anxiety/depression (6.4% vs 5.3%) decreased, whereas reported problems in mobility (5.1% vs 5.9%) and pain/discomfort (9.3% vs 12.6%) increased significantly (*p* < 0.05) in 2013. The regression models revealed a rise (*β* = 1.61, *p* < 0.001) in VAS scores, but a slight drop (*β* = − 0.01, *p* < 0.001) in utility index in 2013 compared with 2008 after controlling for variations in demographic, behavioral, socioeconomic and residential variables. But the effect sizes of the changes over time (estimated by “average change divided by baseline standard deviation”) did not reach the threshold of clinical importance after adjustment for variations in other factors. Higher socioeconomic status (in terms of education, income and residential location) was associated with better EQ-5D-3 L results.

**Conclusion:**

The changing trend (decrease) of the utility index is contradictory to that (increase) of the VAS scores, although neither is deemed clinically important. It is evident that socioeconomic and regional disparities in HRQoL exist in China.

## Introduction

Health-related quality of life (HRQoL) is a multi-dimensional concept that measures self-reported well-being in physical, mental, and social functioning. It captures the impacts of health problems and diseases on quality of life [1–4]. HRQoL has been widely used for assessing outcomes of medical interventions as well as serving as a population health indicator, guiding health policy development and resources allocation [5, 6]. It puts a quality perspective into the measurement of years of life [7, 8]. In combination with traditional health indicators such as mortality and morbidity, a comprehensive indicator (e.g. quality-adjusted life years or QALYs) can be calculated to inform clinical, funding, public policy and management decisions on health products and technologies [9–13].

Over the past few decades, significant progress has been made in measuring HRQoL. The EuroQol Group Five-Dimensional (EQ-5D) series is perhaps one of the most simple and frequently used instruments since early 1990s [14]. The EQ-5D consists of a descriptive system and a visual analogue scale (VAS). The descriptive system comprises five dimensions. Problems related to these dimensions were measured either at three levels (EQ-5D-3 L) or five levels (EQ-5D-5 L) [14]. The combination of reported problems can be converted into a single summary utility index by applying the value sets based on public preferences. The EQ-5D has been recommended by the National Institute for Health and Care Excellence in the UK [8] and the 2011 Guidelines for Pharmacoeconomic Evaluations in China [15].

The EQ-5D-3 L instrument has been validated in Chinese populations [16, 17] and is increasingly being used for assessing HRQoL in the general population [18–20], people with special demographic characteristics [21–25], and people living with chronic conditions [4, 26–34]. Approaches to studies in China using the EQ-5D-3 L evolved over time. The first stage (before 2014) is featured with studies applying the value sets developed in the UK and Japan due to a lack of Chinese value sets [22, 35]. Researchers soon noticed significant differences in public preferences on health status that would result in varied EQ-5D-3 L value sets across nations [36, 37]. In 2014, Liu GG et al. [15] published the EQ-5D-3 L value sets derived from a sample of urban Chinese residents, triggering a wave of second stage studies [20, 38–40]. The localised value sets generated significant different EQ-5D-3 L utility results compared with those derived from the UK and Japanese populations [41]. Unfortunately, the EQ-5D-3 L value sets developed by Liu GG suffer from some major limitations due to the biased sampling: participants were selected conveniently from a few big cities. The authors themselves suggested a need for further refinement of the EQ-5D-3 L value sets in China based on a more inclusive and representative sample [15]. Indeed, there exist great socioeconomic and health disparities between urban and rural populations in China [19]. Public preferences on health status also vary between urban (especially those living in big cities) and rural residents [42]. Such an urban-rural difference in public preferences on health remains even after controlling for variations in socioeconomic status [18]. This highlights the importance of the development of the national representative EQ-5D-3 L value sets in China [15, 43]. In 2018, the national value sets derived from a large representative sample were eventually made available in China [15, 43].

This study aimed to identify changes in the EQ-5D-3 L results over time in China and their determinants. The study was undertaken using the EQ-5D-3 L instead of the EQ-5D-5 L for two reasons. First, two surveys five years apart (2008 and 2013) were available. Over this period of time, China experienced dramatic changes in social and economic development, including reforms of the health system. In this context, changes in HRQoL are likely to occur. Second, the EQ-5D-3 L generates a utility index based on the national value sets derived from public preferences, as well as a VAS indicating individual ratings on their own conditions [44–46]. This enables us to compare changing trends reflected by the two indicators and identify the determinants of contradictions, if any exist.

## Data and methods

### Study design and data collection

#### Data source and sampling method

Data were obtained from the 2008 and 2013 National Health Services Surveys (NHSS), the largest national wide health surveys in China. The NHSS have been conducted every five years since 1993 with coordination from the Centre for Health Statistics and Information under the Ministry of Health [47]. The EQ-5D-3 L has been validated in various populations in China [16–18, 28–34, 48] and incorporated into the NHSS since 2008 [19].

A four-stage stratified cluster probability sampling strategy was adopted to select representative participants in the NHSS [49, 50]. Each stage involved a systematic random sampling approach. In the first stage, urban cities and rural counties (94 in 2008 and 156 in 2013) were selected from the 31 provinces in mainland China in proportion to their population size. In the second stage, five sub-districts/townships in each selected city/county were identified. The third stage narrowed the sample down to two residential communities/villages in each district/township. Finally, 60 households from each residential community/village were invited to participate in the NHSS. All family members in a sampled household were eligible to participate in the NHSS. However, the EQ-5D-3 L was only administered to those who were 15 years or older. This resulted in a final sample size of 120,709 individual respondents in 2008 (excluding 24,921 questionnaires with a proxy respondent and 601 returned questionnaires containing missing values in the EQ-5D-3 L) and 188,720 individual respondents in 2013 (excluding 41,148 questionnaires with a proxy respondent and 196 returned questionnaires containing missing values in the EQ-5D-3 L) for data analyses in this study. The questionnaires with a proxy respondent were excluded because their VAS ratings were unlikely reliable.

#### Questionnaire survey

The questionnaires were administered through face-to-face household interviews conducted by trained local medical workers. The interviewers were rigorously selected considering their qualifications, professional knowledge, sense of responsibility, attitudes, and communication skills. Pre-survey training workshops were offered to all of the interviewers following a standardised protocol. Eligible interviewers had to demonstrate their proper understanding about the purpose of the NHSS and ability to meet data collection standards developed by the Centre for Health Statistics and Information. They also needed to follow pre-defined instructions when problems arose during data collection [49–51].

The face-to-face interviews occurred in the households of participants. Prior to the interviews, the interviewers explained the purpose and procedure of the survey and obtained oral informed consent from each participant. Family members were allowed to serve as a proxy respondent only when the selected participant was younger than 15 years, absent from home, or unable to communicate at the time of the survey. Overall, less than 18% of the returned questionnaire (17% in 2008 and 18% in 2013) were completed by a proxy respondent [50, 52].

A quality assurance team was established in each participating sub-district/township. The returned questionnaires were checked by the quality assurance officers for completeness at the end of each day. Missing data, if any, were addressed through a second interview by the same interviewers. The sampled households were not allowed to be replaced unless three interview attempts failed or the sample size of the residential community/village did not reach 60 [19]. The survey supervisors in each city/county re-interviewed 5% of their local participating households for selected key questions (eight in 2008 and fourteen in 2013) through telephone interviews or field visits. The repeated interviews showed over 95% of consistency with the original ones [49, 52]. The representativeness of the sample was evident in terms of Myer’s Index, DELTA dissimilarity coefficient and GINI concentration ratio for age structure and household size [49, 50, 52, 53].

### Measurements

#### Dependent variables

Three indicators were calculated: percentage of reported problems, utility index and VAS scores.

The descriptive system of EQ-5D comprises five dimensions: mobility (MO), self-care (SC), usual activities (UA), pain/discomfort (PD) and anxiety/depression (AD). This study used the EQ-5D-3 L, measuring problems at three levels: no problem, moderate problem, severe problem.

The combination of reported problems on the five dimensions for each individual was converted into a summarised utility index (*U*). The conversion was based on the value sets developed by Zhuo et al. [43]. It assigns a value to each of the 243 health states classified by the EQ-5D-3 L:

$$ {\displaystyle \begin{array}{l}U=1-\Big({0.0766}^{\ast } MO2+{0.2668}^{\ast } MO3+{0.0441}^{\ast } SC2+{0.2912}^{\ast } SC3+{0.0370}^{\ast } UA2\\ {}+{0.0538}^{\ast } UA3+{0.0274}^{\ast } PD2+{0.0409}^{\ast } PD3+{0.0359}^{\ast } AD2+{0.1771}^{\ast } AD3\Big)\end{array}} $$

where MO2, SC2, UA2, PD2 and AD2 were given a value of 1 if a “moderate problem” presented in the respective dimension, or 0 otherwise. Similarly, MO3, SC3, UA3, PD3 and AD3 were given a value of 1 if an “extreme problem” presented in the respective dimension, or 0 otherwise. This resulted in a possible utility index ranging from 0.1702 to 1.0000 [43].

There exist two value sets in China for the EQ-5D-3 L derived from the Time Trade-Off (TTO) technique: one developed by Liu et al. and another by Zhuo et al. [15, 43]. Both value sets showed smaller disutility for the PD dimension than that from some other countries such as the UK. However, in both cases, linear correlations between the disutility values and the seriousness of health problems remained evident. Some researchers argued that Chinese people, in particular those living in rural areas, are more tolerant to pain problems than the western populations, which may result in smaller disutility for the PD dimension in China [54]. In this study, we chose to adopt the value sets developed by Zhuo and colleagues for several reasons. Firstly, there exist some differences between Liu’s and Zhuo’s value sets. Secondly, Liu’s value sets failed to consider the preference of rural residents in China. But urban-rural disparities in health, social and economic development were still large in China. Finally, Zhuo’s value sets were derived from a nationally representative sample.

The EQ-5D-3 L also contains a VAS, asking respondents to rate their overall health on a scale ranging from ‘worst imaginable health state’ (0) to ‘best imaginable health state’ (100). VAS reflects individual ratings on their own health [18, 19].

#### Independent variables

Changes in HRQoL are determined by a variety of factors at the individual, family, community and society levels [55–57]. There exist several models describing the social and ecological determinants of health [58–62]. In this study, we used the widely cited Dahlgren-Whitehead rainbow model to guide the selection of independent variables. The NHSS covered five broad categories of determinants of health [19, 63]:

**Demographic variables:** Gender and age represent the basic biological feature of respondents.

**Behavioral variables:** The NHSS collected data regarding the current status of smoking (past week), drinking (past 12 months), and physical exercise (past 6 months) of the respondents. Respondents were asked whether and how frequent they had engaged in the aforementioned behaviors at the time of the survey. Respondents were deemed to be physically active if they engaged in regular physical activities at least once a week over the past 6 months.

**Socioeconomic variables:** Socioeconomic status was measured by educational attainment and household income. Respondents were asked to indicate the highest level of school education they had attained. Household income was estimated based on self-reported average income per capita and converted into quintiles (from the lowest 20% to the highest 20%).

**Residential variables:** Respondents were divided into two groups, urban or rural, based on their geographical location in three regions: eastern, central and western. There exist great geographic disparities in socioeconomic development in China. Urban residents enjoy a higher level of entitlement than their rural counterparts. Overall, the eastern coastal region of China is highly developed, whereas the western region lags far behind, with the central region being somewhere in between.

**Time variable:** China’s most recent health system reform started in 2009. The reform achieved great progress in a few years in terms of universal coverage of health insurance and improved access to medical care [49, 64]. We expected that the two rounds of NHSS would capture potential changes resulting from the reform.

### Statistical analyses

We presented the percentages of reported problems on the five dimensions of EQ-5D-3 L. Reported “extreme problems” were rare, and thus were merged with the category of “moderate problems” [38, 47]. Differences in the percentages of reported problems were tested using *χ*^2^ tests. Logistic regression models were established to determine the changes over the two years after controlling for the influence of other independent variables. A *p* value of less than 0.01 was considered statistically significant in line with the Bonferroni adjustment method for multiple comparisons [65].

Means of the utility index and VAS scores were calculated and compared using student *t* tests or analysis of variance (ANOVA). Linear regression models were established to determine changes in VAS scores over the two years after controlling for variations in other independent variables. Because the utility index is bounded to 1, Tobit regression models were established to determine changes in the utility index after controlling for variations in other independent variables [4]. A *p* value of 0.05 was considered statistically significant in the linear and Tobit regression analyses.

The importance of changes in the utility index and VAS scores were also estimated using the indicator of effect size: average change divided by baseline standard deviation (SD), which was proposed by Cohen [66]. The Cohen effect size has now been widely used in identifying the importance of changes: 0.2 small; 0.5 medium; 0.8 large. A medium effect size is usually considered as a difference with clinical meaning [67]. In this study, the medium effect size (0.5) was considered as a threshold for minimal importance of changes in the utility index and VAS scores.

All statistical analyses were performed using STATA 14.0 for Windows.

## Results

### Characteristics of respondents

Slightly less than half of the respondents were male (47.83%). Elderly respondents accounted for less than 20% of respondents (16.45%). More than half of the respondents resided in rural areas (58.79%). Compared with 2008, the 2013 respondents were older (*p* < 0.001), more likely to smoke (*p* < 0.05), drink (*p* < 0.001) and exercise (*p* < 0.001); they also reported higher levels of education (*p* < 0.001). There was a dramatic increase (*p* < 0.001) in the proportion of urban respondents: 49.84% in 2013 compared with 27.71% in 2008 (Table 1).

**Table 1:** Characteristics of respondents

Characteristics	2008		2013		Total		χ^2^	P value
	n	%	n	%	n	%		
Demographic variables								
Gender								
Male	58,169	48.19	89,830	47.60	147,999	47.83	10.26	0.001
Female	62,540	51.81	98,890	**52.40**	161,430	52.17		
Age								
15~44	58,233	48.24	73,522	38.96	131,755	42.58	2600.00	< 0.001
45~64	45,252	37.49	81,532	**43.20**	126,784	40.97		
65~	17,224	14.27	33,666	**17.84**	50,890	16.45		
Behavioral variables								
Smoking								
Yes	30,925	25.73	49,208	**26.09**	80,133	25.95	5.07	0.024
No	89,285	74.27	139,399	73.91	228,684	74.05		
Drinking								
Yes	15,485	13.04	44,005	**23.32**	59,490	19.35	4900.00	< 0.001
No	103,229	86.96	144,706	76.68	247,935	80.65		
Regular physical activity								
Yes	26,831	22.60	55,843	**29.67**	82,674	26.93	1800.00	< 0.001
No	91,896	77.40	132,372	70.33	224,268	73.07		
Socioeconomic variables								
Educational attainment								
Illiterate	18,841	15.62	22,709	12.03	41,550	13.43	2500.00	< 0.001
Primary school	33,630	27.88	48,953	25.94	82,583	26.70		
Junior middle school	43,042	35.69	65,877	34.91	108,919	35.21		
Senior middle school	17,941	14.87	32,435	**17.19**	50,376	16.29		
University/college	7160	5.94	18,746	**9.93**	25,906	8.37		
Household income ranking in local								
Lowest (< 20%)	22,523	18.68	35,702	18.93	58,225	18.83	39.67	< 0.001
20% -	23,369	19.38	35,471	18.80	58,840	19.03		
40%-	24,277	20.13	37,124	19.68	61,401	19.86		
60%-	24,166	20.04	39,084	20.72	63,250	20.45		
Highest (≥80%)	26,256	21.77	41,251	21.87	67,507	21.83		
Residential variables								
Residency								
Rural	87,262	72.29	94,656	50.16	181,918	58.79	15,000.00	< 0.001
Urban	33,447	27.71	94,064	**49.84**	127,511	41.21		
Region								
Eastern	42,305	35.05	66,575	**35.28**	108,880	35.83	1200.00	< 0.001
Central	33,175	27.48	58,306	**30.90**	91,481	30.10		
Western	45,229	37.47	63,839	33.83	103,535	34.07		
Total	120,709	100.00	188,720	100.00	309,429	100.00		

Bold figures indicate a significant increase in 2013 compared with those in 2008 (*p* < 0.05)

### Changes in health-related quality of life

#### Reported health problems

Pain/discomfort was the most frequently reported problem (11.3%), followed by anxiety/depression (5.7%) and mobility (5.6%). Problems in self-care were least reported (3.1%). Compared with 2008, reported problems increased in pain/discomfort (by 3.3 percentage points) and mobility (by 0.7 percentage points) in 2013. By contrast, reported problems in anxiety/depression, self-care and usual activities decreased by 1.2, 0.2 and 0.2 percentage points, respectively (Table 2).

**Table 2:** Reported health problems of respondents

EQ-5D Dimensions	2008		2013		Total		χ^2^	*p*
	n	%	n	%	n	%		
Mobility								
No problem	114,502	94.9	177,667	94.1	292,169	94.4	71.40	< 0.001
Moderate/ Extreme problem	6207	5.1	11,053	**5.9**	17,260	5.6		
Self-care								
No problem	116,774	96.7	182,955	97.0	299,729	96.9	10.20	0.001
Moderate/ Extreme problem	3935	3.3	5765	3.1	9700	3.1		
Usual activities								
No problem	114,881	95.2	179,966	95.4	294,847	95.3	5.89	0.015
Moderate/ Extreme problem	5828	4.8	8754	4.6	14,582	4.7		
Pain/discomfort								
No problem	109,548	90.8	164,920	87.4	274,468	88.7	831.82	< 0.001
Moderate/ Extreme problem	11,161	9.3	23,800	**12.6**	34,961	11.3		
Anxiety/depression								
No problem	112,955	93.6	178,767	94.7	291,722	94.3	180.39	< 0.001
Moderate/ Extreme problem	7754	6.4	9953	5.3	17,707	5.7		

Bold figures indicate a significant increase in 2013 compared with those in 2008 (*p* < 0.01)

#### Utility index and VAS scores

Respondents who were male, younger, richer and had higher educational attainments reported lower health problems than the others, resulting in a higher utility index and VAS scores. Smoking, drinking and regular exercise were associated with less reported health problems and a higher utility index and VAS scores. Compared with rural respondents, urban respondents reported less health problems and consequently had a higher utility index, but they rated lower in VAS (Table 3).

**Table 3:** Reported problems, health utility index and VAS scores stratified by independent variables

Variables	Mobility		Self-care		Usual activities		Pain/discomfort		Anxiety/depression		Mean Utility index (SD)				Mean VAS scores (SD)			
	2008	2013	2008	2013	2008	2013	2008	2013	2008	2013	2008		2013		2008		2013	
Gender																		
male	4.9	5.7	3.1	3.0	4.5	4.5	8.0	11.0	5.7	4.6	0.986	(0.058)	0.986	(0.056)	80.9	(13.8)	81.6	(13.5)
female	5.4	6.0	3.5	3.1	5.1	4.8	10.4	14.1	7.1	5.9	0.985	(0.059)	0.984	(0.056)	79.4	(14.2)	80.3	(13.9)
Age																		
15~44	1.3	1.0	0.8	0.6	1.2	0.9	3.1	3.7	3.1	2.3	0.996	(0.029)	0.986	(0.029)	85.4	(11.3)	86.9	(10.4)
45~64	5.1	4.8	3.0	2.3	4.6	3.6	11.4	14.0	7.5	5.8	**0.985**	(0.057)	**0.986**	(0.050)	**77.5**	(13.7)	**79.3**	(13.3)
65~	18.5	19.0	12.5	10.4	17.5	15.3	24.2	28.7	14.7	10.5	**0.953**	(0.105)	**0.956**	(0.099)	**69.2**	(15.0)	**71.7**	(14.9)
Smoking status																		
Yes	4.2	4.7	2.4	2.2	3.8	3.5	8.2	10.8	5.8	4.5	0.988	(0.049)	0.988	(0.045)	80.6	(13.4)	81.6	(13.0)
No	5.5	6.3	3.5	3.4	5.2	5.1	9.6	13.2	6.7	5.5	0.984	(0.061)	0.984	(0.060)	80.0	(14.2)	80.7	(14.0)
Drinking status																		
Yes	3.3	3.8	1.7	1.5	2.6	2.5	7.3	10.3	4.3	4.1	0.991	(0.039)	0.991	(0.037)	81.2	(12.8)	82.3	(12.5)
No	5.4	6.5	3.5	3.5	5.2	5.3	9.6	13.3	6.8	5.6	0.985	(0.061)	0.983	(0.061)	79.9	(14.2)	80.5	(14.1)
Physical activity																		
Yes	4.0	4.5	2.1	2.0	3.4	3.2	7.6	11.9	4.4	4.3	0.990	(0.040)	0.989	(0.038)	80.3	(13.8)	80.9	(13.1)
No	5.5	6.4	3.6	3.5	5.2	5.3	9.6	12.9	6.9	5.7	0.984	(0.063)	0.983	(0.062)	80.1	(14.1)	80.9	(14.0)
Education status																		
Illiterate	13.2	15.4	9.0	8.9	13.0	13.4	20.6	26.6	14.6	11.7	0.964	(0.090)	0.961	(0.091)	72.4	(15.5)	73.1	(15.4)
Primary school	6.0	8.0	3.8	4.0	5.7	6.1	11.5	17.1	7.5	6.6	0.983	(0.064)	0.980	(0.063)	78.1	(14.1)	78.2	(14.1)
Junior middle school	2.6	3.6	1.5	1.8	2.3	2.8	5.3	9.0	4.0	3.9	0.992	(0.041)	0.990	(0.044)	83.2	(12.5)	83.1	(12.6)
Senior middle school	2.6	2.9	1.4	1.4	2.1	2.2	4.8	7.9	3.3	3.2	0.993	(0.040)	0.992	(0.041)	83.2	(12.7)	83.4	(12.5)
University/college or above	1.9	1.7	1.0	0.8	1.5	1.2	3.7	5.0	2.3	2.5	0.995	(0.031)	0.995	(0.028)	83.6	(12.3)	85.4	(11.2)
Income level																		
Lowest (< 20%)	8.0	9.8	5.2	5.3	7.7	8.1	12.5	18.3	9.9	10.0	0.977	(0.074)	0.975	(0.072)	77.0	(15.5)	77.7	(15.6)
20% -	5.2	6.1	3.3	3.2	4.8	4.8	9.5	13.0	6.8	5.7	0.985	(0.060)	0.984	(0.058)	79.8	(14.1)	80.5	(13.9)
40%-	4.9	5.0	3.0	2.6	4.4	3.9	8.7	11.7	6.0	4.6	0.987	(0.055)	0.987	(0.052)	80.5	(13.8)	81.6	(13.3)
60%-	4.0	4.6	2.5	2.3	3.9	3.5	7.8	10.7	5.0	4.2	0.989	(0.051)	0.988	(0.049)	81.2	(13.4)	81.9	(12.9)
Highest (≥80%)	3.9	4.3	2.6	2.2	3.7	3.3	8.0	10.0	5.0	3.8	0.989	(0.049)	0.989	(0.047)	81.7	(13.1)	82.4	(12.6)
Residency																		
Urban	4.7	5.7	2.8	2.8	4.8	4.3	9.3	12.5	6.4	5.0	0.987	(0.055)	0.985	(0.055)	79.3	(14.0)	80.6	(13.8)
Rural	5.3	6.0	3.5	3.3	5.2	5.0	9.8	12.8	7.0	5.6	0.985	(0.060)	0.984	(0.058)	80.4	(14.1)	81.2	(13.7)
Region																		
Eastern	4.4	5.4	2.7	2.8	4.0	4.2	8.2	11.1	4.7	4.2	0.988	(0.053)	0.986	(0.054)	81.7	(13.6)	82.1	(13.2)
Central	5.1	6.0	3.3	3.1	4.8	4.6	8.9	13.4	5.1	5.5	0.986	(0.061)	0.984	(0.058)	79.9	(14.0)	80.6	(14.0)
Western	5.9	6.2	3.7	3.2	5.6	5.1	10.5	13.5	9.0	5.5	**0.983**	(**0.061**)	**0.984**	(**0.057**)	**78.8**	(**14.4**)	**79.9**	(**13.9**)
Total	5.1	5.9	3.3	3.1	4.8	4.6	9.3	12.6	6.4	5.3	0.985	(0.058)	0.985	(0.056)	80.1	(14.1)	80.9	(13.7)

Significant changes (*p* < 0.05) between 2008 and 2013 were found in all indicators; Bold figures illustrate consistent changes (increase) in the utility index and VAS scores over the two years

Overall, the respondents had an average utility index around 0.985 and an average VAS score around 80. Compared with 2008, there was a significant decrease (*p* < 0.001) in the utility index, but an increase (*p* < 0.001) in VAS scores in 2013, albert in very small effect sizes (− 0.01 and 0.06, respectively). However, some subgroups stratified by age and region defied the overall trends, showing an increase in both the utility index and VAS scores (Table 3). Nonetheless, neither changes in the utility index (effect sizes ranging from − 0.31 to 0.03) nor changes in VAS scores (effect sizes ranging from − 0.01 to 0.17) reached the threshold of minimal clinical importance (Table 3).

### Findings of multivariate regression models

Changes in reported health problems, the utility index and VAS scores over time remained significant after controlling for the influence of other independent variables.

#### Factors associated with reported health problems

The logistic regression models confirmed that there was an increased likelihood of reporting problems in 2013 in pain/discomfort (AOR = 1.34) and mobility (AOR = 1.07), but a decreased likelihood of reporting problems in self-care (AOR = 0.90), usual activity (AOR = 0.92), and anxiety/depression (AOR = 0.80) compared with 2008 (Table 4).

**Table 4:** Multiple logistic regression analyses on reported health problems

Independent variable	Mobility			Self-care			Usual activity			Pain/discomfort			Anxiety/depression		
	AOR	95%CI		AOR	95%CI		AOR	95%CI		AOR	95%CI		AOR	95%CI	
Gender (Male as reference)															
Female	0.77**	0.74	0.80	0.69**	0.65	0.72	0.71**	0.68	0.74	1.18**	1.14	1.21	1.05*	1.01	1.10
Age (< 45 years as reference)															
45~64	4.14**	3.90	4.40	3.57**	3.30	3.87	3.62**	3.39	3.86	3.71**	3.58	3.85	2.37**	2.27	2.48
65~	15.95**	14.97	17.00	14.49**	13.33	15.74	13.89**	12.98	14.86	7.98**	7.66	8.31	4.00**	3.80	4.21
Smoking (Yes as reference)															
No	1.35**	1.29	1.42	1.51**	1.42	1.61	1.45**	1.37	1.52	1.08**	1.05	1.12	1.10**	1.05	1.15
Drinking (Yes as reference)															
No	1.60**	1.52	1.69	2.21**	2.05	2.38	1.98**	1.87	2.10	1.14**	1.10	1.18	1.29**	1.23	1.36
Regular physical activity (Yes as reference)															
No	1.61**	1.53	1.69	1.96**	1.83	2.09	1.74**	1.65	1.83	1.12**	1.09	1.16	1.30**	1.24	1.36
Educational attainment (Illiterate as reference)															
Primary school	0.72**	0.69	0.75	0.67**	0.64	0.71	0.66**	0.63	0.69	0.79**	0.76	0.81	0.69**	0.66	0.72
Junior middle school	0.55**	0.52	0.58	0.52**	0.48	0.55	0.49**	0.47	0.52	0.57**	0.55	0.59	0.55**	0.52	0.58
Senior middle school	0.46**	0.43	0.49	0.43**	0.40	0.48	0.41**	0.38	0.45	0.48**	0.46	0.51	0.48**	0.45	0.51
University/college or above	0.35**	0.32	0.39	0.34**	0.29	0.39	0.31**	0.27	0.35	0.41**	0.38	0.44	0.44**	0.40	0.49
Household income ranking in local (< 20% as reference)															
20%-	0.76**	0.73	0.80	0.77**	0.73	0.82	0.74**	0.71	0.78	0.80**	0.77	0.83	0.74**	0.71	0.78
40%-	0.69**	0.66	0.73	0.72**	0.67	0.76	0.67**	0.64	0.71	0.75**	0.72	0.78	0.65**	0.62	0.68
60%-	0.63**	0.60	0.66	0.65**	0.61	0.70	0.63**	0.59	0.66	0.68**	0.66	0.71	0.58**	0.55	0.61
Highest (≥80%)	0.63**	0.60	0.66	0.68**	0.64	0.73	0.63**	0.60	0.67	0.67**	0.65	0.70	0.57**	0.54	0.60
Residency (Urban as reference)															
Rural	0.84**	0.80	0.87	0.87**	0.83	0.92	0.91**	0.87	0.95	0.93**	0.90	0.96	0.96*	0.92	1.00
Region (Eastern as reference)															
Central	1.17**	1.13	1.22	1.20**	1.14	1.27	1.17**	1.12	1.23	1.23**	1.19	1.27	1.26**	1.21	1.32
Western	1.29**	1.24	1.34	1.29**	1.22	1.35	1.34**	1.29	1.40	1.35**	1.31	1.39	1.75**	1.68	1.81
Year (2008 as reference)															
2013	1.07**	1.04	1.11	0.90**	0.86	0.94	0.92**	0.89	0.96	1.34**	1.31	1.38	0.80**	0.77	0.83

* p < 0.05, * *p < 0.01

#### Factors associated with the utility index and VAS scores

The multiple linear and Tobit regression models confirmed the contradictory changing trends over time: decreased (*β* = − 0.01, *p* < 0.001) utility index vs increased (*β* = 1.61, p < 0.001) VAS scores, after adjustment for variations in the other independent variables. But the effect sizes of those changes over time did not reach the threshold of minimal clinical importance (Table 5). Age, education, income and residential location were significant predictors of the utility index and VAS scores. Their effect sizes reached the threshold of minimal clinical importance for the utility index. But for VAS scores, only the age effect reached the threshold of minimal clinical importance.

**Table 5:** Multiple linear and Tobit regression analyses on the health utility index and VAS scores

Independent variable	Utility index						VAS scores					
	β	SE	p	95%CI		Effect sizes	β	SE	p	95%CI		Effect sizes
Gender (Male as reference)												
Female	0.00	0.00	0.311	−0.00	0.00	0.03	−0.55	0.06	< 0.001	− 0.66	− 0.44	−0.04
Age (< 45 years as reference)												
45~64	−0.11	0.00	< 0.001	−0.11	− 0.10	−1.92	−7.01	0.05	< 0.001	−7.11	−6.91	− 0.51
65~	−0.21	0.00	< 0.001	−0.21	− 0.21	−3.74	− 13.59	0.08	< 0.001	− 13.75	− 13.43	− 0.99
Smoking (Yes as reference)												
No	− 0.02	0.00	< 0.001	− 0.02	− 0.01	− 0.28	− 0.18	0.06	0.006	− 0.30	− 0.05	− 0.01
Drinking (Yes as reference)												
No	− 0.02	0.00	< 0.001	− 0.03	− 0.02	− 0.40	−0.95	0.06	< 0.001	− 1.08	− 0.83	− 0.07
Regular physical activity (Yes as reference)												
No	−0.02	0.00	< 0.001	−0.03	−0.02	− 0.43	−0.50	0.06	< 0.001	−0.62	− 0.39	− 0.04
Educational attainment (Illiterate as reference)												
Primary school	0.03	0.00	< 0.001	0.03	0.04	0.61	2.39	0.09	< 0.001	2.21	2.56	0.17
Junior middle school	0.07	0.00	< 0.001	0.06	0.07	1.16	4.46	0.09	< 0.001	4.29	4.64	0.33
Senior middle school	0.08	0.00	< 0.001	0.08	0.09	1.44	5.11	0.10	< 0.001	4.91	5.31	0.37
University/college or above	0.09	0.00	< 0.001	0.09	0.10	1.68	5.25	0.12	< 0.001	5.02	5.48	0.38
Household income ranking in local (< 20% as reference)												
20%-	0.03	0.00	< 0.001	0.02	0.03	0.50	1.69	0.08	< 0.001	1.54	1.85	0.12
40%-	0.04	0.00	< 0.001	0.04	0.04	0.69	2.36	0.08	< 0.001	2.21	2.51	0.17
60%-	0.05	0.00	< 0.001	0.04	0.05	0.84	2.78	0.08	< 0.001	2.63	2.93	0.20
Highest (≥80%)	0.05	0.00	< 0.001	0.04	0.05	0.86	3.22	0.08	< 0.001	3.07	3.37	0.23
Residency (Urban as reference)												
Rural	0.01	0.00	< 0.001	0.01	0.02	0.26	1.65	0.05	< 0.001	1.54	1.76	0.12
Region (Eastern as reference)												
Central	−0.02	0.00	< 0.001	−0.02	−0.01	−0.31	−1.73	0.06	< 0.001	−1.84	−1.62	−0.13
Western	−0.03	0.00	< 0.001	−0.03	− 0.03	− 0.56	−2.71	0.05	< 0.001	−2.82	−2.61	− 0.20
Year (2008 as reference)												
2013	−0.01	0.00	< 0.001	−0.01	−0.01	− 0.20	1.61	0.05	< 0.001	1.51	1.70	0.12

## Discussion

Changes in HRQoL over time in the Chinese populations between 2008 and 2013 are minimal, if ever exist, according to this study. Compared with 2008, reported problems in mobility and pain/discomfort increased in 2013, but reported problems in anxiety/depression, self-care and usual activities decreased. The mixture of changing health problems resulted in a small decline in utility index. However, VAS scores defied the trend, showing a slight increase over time. It is worth noting that the time changes in both indicators were small and failed to reach the threshold of clinical importance in this study. Further studies are needed to verify the contradictory changing trends and their policy and clinical implications. There is evidence that these two indicators measure quite different constructs of HRQoL [68]. VAS scores capture real-time individual ratings, whereas the utility index applies past population preferences to value the current health status. Certain populations (for example women) may have higher than average expectations on health, leading to relatively lower ratings on VAS [18, 19].

Age is a significant predictor of HRQoL. Older people are more likely to experience health problems than their younger counterparts as indicated in this study and others [4, 6, 18, 20, 45, 69–75], resulting in significant lower utility index and VAS scores. The effect sizes of age are the largest among all of the independent variables assessed. This indicates a great challenge to China as its population is ageing rapidly. Previous studies reported consistent lower utility index and VAS scores in women compared with men [4, 6, 45, 71–73, 75–77]. But interestingly, gender differences in both the utility index and VAS scores were found to be too small to be deemed clinically important in this study and there is no evidence to support a claim of lower utility index in women. In line with in studies conducted elsewhere in China [78–80], this study also provides additional evidence on the associations between behavioral factors (smoking, drinking, and exercise) and HRQoL.

Socioeconomic disparities in HRQoL deserve a serious concern in China. Low socioeconomic status appeared to be associated with lower HRQoL regardless of which indicator was applied in this study. Those who were wealthier and had higher educational attainments had higher scores in both utility and VAS scores. In line with regional disparities in socioeconomic development in China, higher scores in utility and VAS scores were also found for those who resided in the more developed eastern region. These results are consistent with findings of other studies [4, 6, 18–20, 45, 69–72, 74, 81]. Between 2008 and 2013, China’s wealth increased exponentially, which was accompanied by dramatic improvement of people’s living standards. Life expectancy increased from 74.8 to 76.3 years over the period of time [82]. Unfortunately, this development had not translated into a higher HRQoL as indicated in the crude utility index and VAS scores results.

China experienced rapid urbanisation process over the past few decades. But rural residents still comprised 46.27% of the total population in 2013 [82]. This study adopted the value sets derived from a sample inclusive of rural residents. It showed that rural residents reported more health problems compared with their urban counterparts, but they had higher scores in the utility index and VAS scores after controlling for other factors. Clearly, urbanisation is unlikely to offer a silver bullet to improving the HRQoL of Chinese people. Future studies should explore whether rural residents in China hold different perceptions and values on health problems. Many young people in rural and undeveloped regions chose to leave their villages, hoping to live a better life [22, 24, 83–85]. This may have significant implications on both individual ratings and population preferences on health.

### Limitations

There are several limitations in this study. Although the study sample is large and representative of the nation, we do not have control over the choice of data and questions definitions [18, 19]. The two rounds of cross-sectional surveys were not undertaken using the same pool of participants [65, 86], which prevents us from drawing casual conclusions. In addition, the EQ-5D-3 L used in the NHSS has more significant ceiling effects than the EQ-5D-5 L. However, the comparison of the two large national cross-sectional surveys provides valuable indications on the overall changing trends of HRQoL, which is important to inform future studies.

## Conclusions

The changing trend (decrease) of the utility index is contradictory to that (increase) of the VAS in China over the period between 2008 and 2013, although neither is deemed clinically important. Such a change (or lack of) cannot be fully explained by the changing demographic and socioeconomic status in China. Further studies are warranted to explore the underlining reasons, in particular the role of health reforms [64]. Meanwhile, it is important to note that socioeconomic and regional disparities in HRQoL are increasing in China. The rapid socioeconomic transition and demographic changes in China may also have some potential impacts on public preferences on health status.

## Abbreviations

AD: Anxiety/depression
ANOVA: Analysis of Variance
AOR: Adjusted Odds Ratio
EQ-5D: European Quality of Life Five Dimensions
EQ-5D-3 L: 3-level EQ-5D
EQ-5D-5 L: 5-level EQ-5D
HRQoL: Health-Related Quality of Life
MO: Mobility
NHSS: National Health Services Surveys
PD: Pain/discomfort
QALYs: Quality-Adjusted Life Years
SC: Self-care
SD: Standard Deviation
TTO: Time Trade-Off
UA: Usual Activities
VAS: Visual Analogue Scale
